# Recall by genotype and cascade screening for familial hypercholesterolemia in a population-based biobank from Estonia

**DOI:** 10.1038/s41436-018-0311-2

**Published:** 2018-10-01

**Authors:** Maris Alver, Marili Palover, Aet Saar, Kristi Läll, Seyedeh Maryam Zekavat, Neeme Tõnisson, Liis Leitsalu, Anu Reigo, Tiit Nikopensius, Tiia Ainla, Mart Kals, Reedik Mägi, Stacey B. Gabriel, Jaan Eha, Eric S. Lander, Alar Irs, Anthony Philippakis, Toomas Marandi, Pradeep Natarajan, Andres Metspalu, Sekar Kathiresan, Tõnu Esko

**Affiliations:** 1 Estonian Genome Center, Institute of Genomics, University of Tartu, Tartu, Estonia; 2 Department of Biotechnology, Institute of Molecular and Cell Biology, University of Tartu, Tartu, Estonia; 3 Department of Cardiology, Institute of Clinical Medicine, University of Tartu, Tartu, Estonia; 4 Cardiology Centre, North Estonia Medical Centre, Tallinn, Estonia; 5 Institute of Mathematics and Statistics, University of Tartu, Tartu, Estonia; 6 Broad Institute of Harvard and MIT, Cambridge, MA USA; 7 Yale School of Medicine, New Haven, CT USA; 8 Department of Clinical Genetics in Tallinn, United Laboratories, Tartu University Hospital, Tartu, Estonia; 9 Heart Clinic, Tartu University Hospital, Tartu, Estonia; 10 Cardiovascular Research Center and Center for Genomic Medicine, Massachusetts General Hospital, Boston, MA USA; 11 Department of Medicine, Harvard Medical School, Boston, MA USA

**Keywords:** recall by genotype, population-based biobank, familial hypercholesterolemia, cascade screening, genomics-guided disease management

## Abstract

**Purpose:**

Large-scale, population-based biobanks integrating health records and genomic profiles may provide a platform to identify individuals with disease-predisposing genetic variants. Here, we recall probands carrying familial hypercholesterolemia (FH)-associated variants, perform cascade screening of family members, and describe health outcomes affected by such a strategy.

**Methods:**

The Estonian Biobank of Estonian Genome Center, University of Tartu, comprises 52,274 individuals. Among 4776 participants with exome or genome sequences, we identified 27 individuals who carried FH-associated variants in the *LDLR*, *APOB*, or *PCSK9* genes. Cascade screening of 64 family members identified an additional 20 carriers of FH-associated variants.

**Results:**

Via genetic counseling and clinical management of carriers, we were able to reclassify 51% of the study participants from having previously established nonspecific hypercholesterolemia to having FH and identify 32% who were completely unaware of harboring a high-risk disease-associated genetic variant. Imaging-based risk stratification targeted 86% of the variant carriers for statin treatment recommendations.

**Conclusion:**

Genotype-guided recall of probands and subsequent cascade screening for familial hypercholesterolemia is feasible within a population-based biobank and may facilitate more appropriate clinical management.

## Introduction

With the plummeting costs of exome (ES) and genome sequencing (GS), the collection of high-coverage genomic data is increasingly becoming routine in genetic research. Population-based biobanks, which combine such genomic data with electronic health records (EHRs) and clinical phenotyping, provide an opportunity to study the population-specific landscape of clinically important phenotypes and enable recall by genotype (RbG) studies of individuals carrying genetic variants of interest, as well as their relatives.^1^ The genotype-first approach (i.e., genetic variant ascertainment precedes phenotypic measurement) has been established as an effective strategy in human genetics, allowing the refinement of (endo)phenotypes and study of biological heterogeneity by overcoming initial phenotypic ascertainment biases.^2,3^ Applying such strategy within a health care–associated biobank enables to additionally investigate whether the RbG approach benefits clinical disease management.

Familial hypercholesterolemia (FH) is as an ideal case in which to implement such a strategy, being one of the most common single-gene disorders (prevalence of 1 in 217 in Europe^4^) and having actionable treatment options.^5^ FH, which primarily results from deleterious variants in the *LDLR*, *APOB*, or *PCSK9* genes, involves the dysfunction of the low-density lipoprotein (LDL) receptor and concomitant overactivity of 3-hydroxy-3-methyl-glutaryl-coenzyme A (HMG-CoA) reductase, resulting in elevated low-density lipoprotein cholesterol (LDL-C) levels and a substantially increased risk of premature coronary heart disease (CHD).^6,7^ While severely high LDL-C concentrations (>4.9 mmol/L) are commonly acknowledged in clinical FH diagnosis, recent studies highlight that FH-associated variant carriers display a wide spectrum of LDL-C levels, and the risk for CHD persists across the range of these values.^8,9^

The Estonian Genome Center’s population-based collection of comprehensive health data and genomic profiles on more than 50,000 individuals offers a valuable data source in which to assess the effectiveness of genotype-guided clinical management. Here, we apply the RbG approach coupled with cascade screening, genetic counseling, and deep-phenotyping within the Estonian population-based biobank to FH, a condition that is linked to significant morbidity and mortality but is substantially modifiable when recognized and managed.

## Materials and methods

### Cohort overview

The Estonian Biobank is a population-based biobank of the Estonian Genome Center at the University of Tartu (EGCUT). The cohort of 52,274 individuals aged 18 and over closely reflects the age, sex, and geographical distribution of the Estonian population. All participants have signed a broad informed consent form, which allows the continuous updating of epidemiologic data through periodical linking to national electronic databases and recontacting of participants. Detailed overview of the biobank has been described previously.^10^ High-coverage sequencing data was available for 10% of the cohort. The genomes of 2535 individuals, selected randomly by county of birth to represent as much of the genetic diversity as possible, were sequenced at the Broad Institute (Cambridge, MA, USA). Exomes of an additional subset of 2500 individuals were sequenced at Nestlé Institute of Health Sciences (Lausanne, Switzerland). The latter sample set contained 878 healthy constitutionally thin individuals (body mass index [BMI] in the lowest 4% quantile at EGCUT after adjustment for age and sex) and 1622 healthy BMI controls (BMI in 30–50% quantile at EGCUT after adjustment for age and sex) selected from the Estonian Biobank within the age range of 20–45 years. GS followed a polymerase chain reaction (PCR)-free sample preparation and was sequenced on the Illumina HiSeq X Ten using 150-bp paired-end reads with mean coverage of 30×. ES samples were sequenced using the Agilent SureSelect Human All Exon V5+ UTRs Kit according to the manufacturer’s recommendations with mean target coverage of 67×. Sequenced reads were aligned against the GRCh37/hg19 human reference genome using BWA-MEM^11^ v0.7.7, and sequence variants were annotated with Variant Effect Predictor^12^ version 87 (Gencode v19 on assembly GRCh37.p13) and ANNOVAR.^13^ Baseline lipid parameters for the GS subset were measured with either a conventional enzymatic colorimetric method at Tartu University Hospital or North Estonia Medical Centre (*n* = 1025; measured in mmol/L), or with Vertical Auto Profile (VAP) based on density gradient ultracentrifugation by Atherotech^14,15^ (*n* = 2181; measured in mg/dl), and for the ES subset with the conventional method only. The effect of statin treatment in individuals who had self-reported use at baseline lipid measurement was taken into account by dividing LDL-C value by 0.7, as implemented previously^16^ (termed “statin-adjusted”) (Table S1).

The project was approved by the Research Ethics Committee of the University of Tartu (application number 253/T-14, December 2015 and 234/T-12, March 2014) and complies with the Declaration of Helsinki.

### Identification of FH-associated variants

Rare (minor allele frequency [MAF] <0.5%) deleterious variants in three FH-associated autosomal dominant genes (*LDLR*, *APOB*, *PCSK9*) were ascertained in carriers with untreated baseline LDL-C level of ≥4.0 mmol/L within the GS and ES data sets. The determined threshold corresponds to the lowest LDL-C value considered in the Dutch Lipid Clinic Network (DLCN) diagnostic criteria for familial hypercholesterolemia diagnosis,^17^ suggested by the European Society of Cardiology and European Atherosclerosis Society for the management of dyslipidemias^18^ (Table S2).

We considered variants annotated as loss-of-function (i.e., premature stop codon, disruption of an essential splice site, or frameshift of the reading frame), deleterious missense variants in *LDLR*, gain-of-function missense variants in *PCKS9*, and deleterious variants in exon 26 (encompassing the binding site of the LDL receptor)^19^ in *APOB*. A variant was considered as FH-associated if MAF was <0.5% and  identified in a carrier with baseline LDL-C level of ≥4.0 mmol/L, and was (1) reported as pathogenic/likely pathogenic in NCBI-ClinVar^20^ and/or (2) determined to be pathogenic according to in silico prediction algorithms.

### Management of probands and cascade screening

Carriers of the identified FH-associated variants (probands) were contacted via regular mail and upon positive response scheduled for an initial appointment with a clinical genetics specialist and clinical cardiologist either at Tartu University Hospital or North Estonia Medical Centre. At the initial appointment, family and medical history was specified, and a standard clinical examination including ascertainment of features specific to FH was performed. Then, 50 ml of fasting blood from a peripheral vein was drawn for biochemical measurements (including enzymatic colorimetric assay–based lipid measurements) and for a DNA-based confirmation of the genetic finding. If the proband did not have clinical atherosclerotic cardiovascular disease (ASCVD), investigations for subclinical atherosclerosis were performed (computed tomography for coronary artery calcium [CAC] score, carotid ultrasound for intima-media thickness [IMT] assessment, and exercise electrocardiogram [ECG]). Subclinical ASCVD was determined as CAC (Agatston score) >0, or presence of atherosclerotic plaque in carotid artery (focal wall thickening >50% greater than the surrounding vessel wall or focal region with an IMT measurement ≥1.5 mm protruding into the lumen). After the confirmation of the identified variant by Sanger sequencing at a CLIA-certified laboratory, a feedback appointment was scheduled. During the visit, the specified genetic finding was disclosed, the pathophysiology of FH and the pattern of inheritance together with the probability for close relatives being affected was explained, and a summary of the clinical and imaging investigations and treatment or changes in treatment, if necessary, were provided. The investigation of the first and second degree relatives invited to participate in cascade screening followed the same approach as for the probands. Only those individuals who carried the variants identified in the family were subjected to instrumental investigations. For extended details on cascade screening please refer to Supplementary Materials and Methods and Figure S1.

### LDL-C level association analysis

To investigate the effect of FH-associated genetic variants on LDL-C levels, a subset of 978 FH-associated variant noncarriers from the GS sample set with available conventionally measured LDL-C levels was formed. FH-associated variant carriers included 21 probands and 20 relatives who participated in the study and had LDL-C measurements available at the initial appointment. Statin treatment was taken into account by dividing LDL-C value by 0.7 (ref. ^20^) (termed “statin-adjusted”). A mixed linear model (package lme4qtl)^21^ in R (R Project for Statistical Computing^22^) version 3.4.1 was used to analyze the association between LDL-C levels and FH-associated variants: the presence of any FH-associated variant was defined as a binary variable in all samples, and the model was adjusted for age, age^2^ and sex, with relatedness between individuals taken into account as a kinship matrix. While the kinship matrix for FH-linked variant noncarriers (*n* = 978) and index cases (*n* = 21) was calculated based on genome-wide genetic data, the kinship of family members (*n* = 20) was added such that the relatedness with FH-linked noncarriers was deemed zero, and with the respective family members as the coefficient of relationship based on the pedigree overview. Because the significance effect is not reported in the abovementioned package, it was estimated on a sample set, where up to third degree relatives had been removed (3 probands, all cascades, and 53 noncarriers were excluded).

### CAC score sensitivity analysis

The CAC scores of FH-associated variant carriers with subclinical disease and CAC >0 (*n* = 19) were compared with the distribution of CAC scores in the Multi-Ethnic Study of Atherosclerosis (MESA) subcohort according to the CAC Score Reference Values.^23^ The respective MESA cohort consisted of 2503 men and women of Caucasian ethnicity who were free of symptomatic clinical ASCVD and treated diabetes. While the CAC scores at the 25th and the 75th percentiles were available for the age range of 45 to 84, the estimated percentiles for those <45 years of age were set at zero.

Please refer to Supplementary Material and Methods for expanded Methods section.

## Results

Upon screening for rare deleterious genetic variants in GS (*n* = 2420) and ES (*n* = 2356) samples, we identified 27 probands who carry a total of 11 distinct heterozygous variants in FH-associated genes (*LDLR*, *APOB*, *PCSK9*) (Table S3). All variants were confirmed with Sanger sequencing prior to the call-back of probands with nonconcordance rate of zero, indicating high reliability of deep-coverage GS/ES for variant identification.

### Study participants

**Fig. 1:**
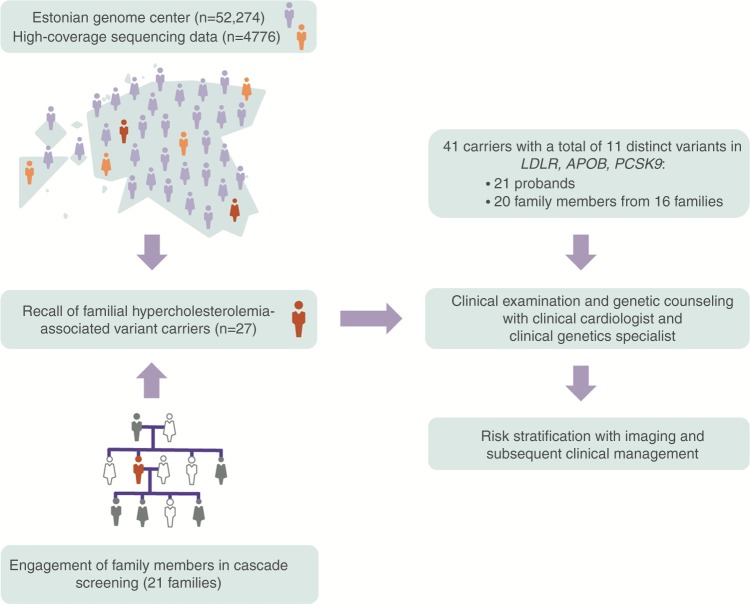
**Overview of recall by genotype approach for familial hypercholesterolemia within the Estonian Genome Center and subsequent clinical management.**

Twenty-one (78%) of the 27 FH-associated variant carriers (mean age 47.1 [SD 15.9], 43% were female) responded positively to the call-back and were scheduled for an appointment with a clinical cardiologist and clinical genetics specialist. Six individuals declined to participate either due to health issues or missing contact information (Figure S1). Of 21 participants, 10 probands harbored a missense variant in *APOB* (p.Arg3527Gln), 10 carried a total of eight distinct missense variants in *LDLR*, and 1 had a missense variant in the *PCSK9* gene (Table S3). After clinical management and genetic counseling, all carriers were guided to engage their first and second degree relatives in cascade screening. Of 112 invited family members, 64 relatives (57%, mean age 46.6 [SD 17.1], 52% were female) participated with at least one cascade joining from 16 families. Twenty relatives (mean age 47.3 [SD 17.5], 55% were female) were heterozygous for the FH-associated variant, yielding one new case per proband. Altogether, 41 carriers were identified via RbG and cascade screening, demonstrating high recontact rate of probands (78%) and successful engagement of family members (76% of the invited families engaged) (Fig. 1).

### Clinical status of FH-associated variant carriers

Prior to participating, among 41 FH-associated variant carriers, 21 (51%) had nonspecific hypercholesterolemia diagnosed or increased LDL-C level identified, 3 (7%) had a clinical FH diagnosis, and 4 (10%) had ASCVD manifested. Seventeen individuals (41%) did not carry diagnoses for hypercholesterolemia or ASCVD. Of 41 FH-associated variant carriers, 6 individuals (15%) reported positive family history of premature ASCVD in a first degree family member. While 19 FH-associated variant carriers (46%) had statins prescribed, 13 (32%) of the 19 carriers reported statin use and 1 was taking over-the-counter lipid-lowering supplements at the initial appointment. LDL-C levels varied from 3.22 mmol/L to 11.56 mmol/L (mean 5.78 mmol/L [SD 2.05]) among carriers not on treatment (*n* = 28). The mean statin-adjusted LDL-C concentration in carriers on treatment (*n* = 13) was 5.79 mmol/L (SD 1.41), ranging from 3.89 mmol/L to 9.10 mmol/L (Table 1). None of those on treatment had achieved the target of LDL-C <2.6 mmol/L recommended for FH cases without ASCVD according to the ESC guidelines^18^ (Table S5). Only two carriers (5%) had a visible physical sign (arcus cornealis) of FH (77- and 78-year-old brother and sister carrying *APOB* p.Arg3527Gln).

**Table 1:** Characteristics of study participants

Characteristics	Probands	Participating family members	
	Participants	Carriers	Noncarriers
*N*	21	20	44
Age (years)	47.7 (15.9)	47.3 (17.5)	46.25 (17.11)
Women	9 (43%)	9 (45%)	22 (50%)
Blood pressure (mmHg)			
Systolic	132.4 (25.0)	135.2 (19.2)	137.6 (22.1)
Diastolic	82. 9 (10.2)	84.8 (12.1)	84.5 (11.3)
LDL-C (mmol/L)	5.3 (2.1)	5.1 (1.8)	3.4 (0.9)
TC (mmol/L)	7.3 (2.2)	6.8 (1.9)	5.2 (0.9)
HDL-C (mmol/L)	1.9 (0.8)	1.6 (0.4)	1.7 (0.4)
TG (mmol/L)	1.2 (0.7)	1.4 (0.9)	1.1 (0.7)
Diabetes mellitus	0 (0%)	1 (5%)	3 (7%)
Hypercholesterolemia	13 (62%)	8 (40%)	3 (7%)
FH	3 (14%)	0 (0%)	0 (0%)
Prevalent ASCVD	2 (10%)	2 (10%)	6 (14%)
Body mass index	26.5 (9.2)	27.8 (5.7)	27.3 (5.3)
Current smokers	7 (33%)	4 (20%)	13 (30%)
Lipid-lowering medication prescription	13 (62%)	6 (30%)	8 (18%)
Lipid-lowering medication use	8 (38%)	5 (25%)	9 (20%)
Antihypertensive medication use	4 (19%)	6 (30%)	13 (30%)

Data are number (%) or mean (SD).

*ASCVD* atherosclerotic cardiovascular disease, *FH* familial hypercholesterolemia, *HDL* high-density lipoprotein, *LDL* low-density lipoprotein, *TC* total cholesterol, *TG* triglycerides.

**Fig. 2:**
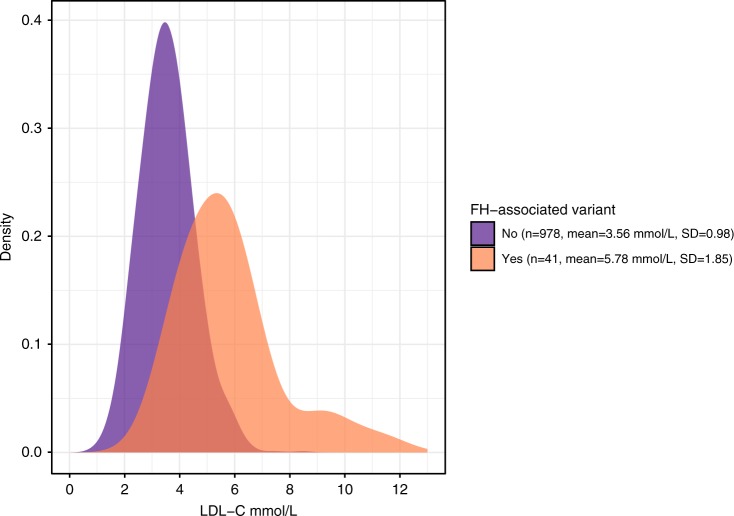
**Distribution of statin-adjusted LDL-C values in FH-associated variant carriers.** (*n* = 41, mean LDL-C 5.78 mmol/L, SD 1.85) and noncarriers (*n* = 978, mean LDL-C 3.56 mmol/L, SD 0.98). The LDL-C level was increased by 2.33 mmol/L (SD 0.18) in individuals harboring an FH-associated variant, compared with noncarriers. *FH* familial hypercholesterolemia, *LDL* low-density lipoprotein.

Compared with 978 noncarriers in the GS sample set with conventionally measured LDL-C levels, the LDL-C value was increased on average by 2.33 mmol/L (SD 0.18, adjusted model *p* = 1.55 × 10^−21^) in FH-associated variant carriers (*n* = 41). *APOB* variant carriers had on average greater LDL-C concentrations (*n* = 20, *β* = 2.96 mmol/L, SD 0.25) than individuals with a variant in *LDLR* (*n* = 20, *β* = 1.73 mmol/L, SD 0.24), compared with noncarriers. LDL-C level of a *PCSK9* variant carrier was 1.5 SD higher than in noncarriers. Despite higher LDL-C levels among FH-associated variant carriers, we observed substantial overlap in the LDL-C distributions between carriers and noncarriers (Fig. 2), highlighting current clinical screening challenges. Furthermore, low statin use and LDL-C levels above treatment goals in FH-associated variant carriers indicate lack of genetic and clinical awareness of the disease.

### Imaging-based phenotyping

To determine the presence of subclinical cardiovascular pathology, all probands and carrier relatives without prevalent ASCVD underwent imaging-based phenotyping (carotid ultrasound and computed tomography). While 4 FH-associated variant carriers (10%) had clinically manifested ASCVD (mean age 73.5 [SD 11.6], 50% were female) and 3 (7%) declined the procedures, 20 FH-associated variant carriers (49%) displayed subclinical disease (mean age 50.5 [SD 12.8], 46% were female, mean statin-adjusted LDL-C 6.31 [SD 1.76]) and 14 (34%) did not (mean age 36.2 [SD 8.9], 43% were female, mean statin-adjusted LDL-C 4.55 [SD 1.06]) (Table S5).

**Fig. 3:**
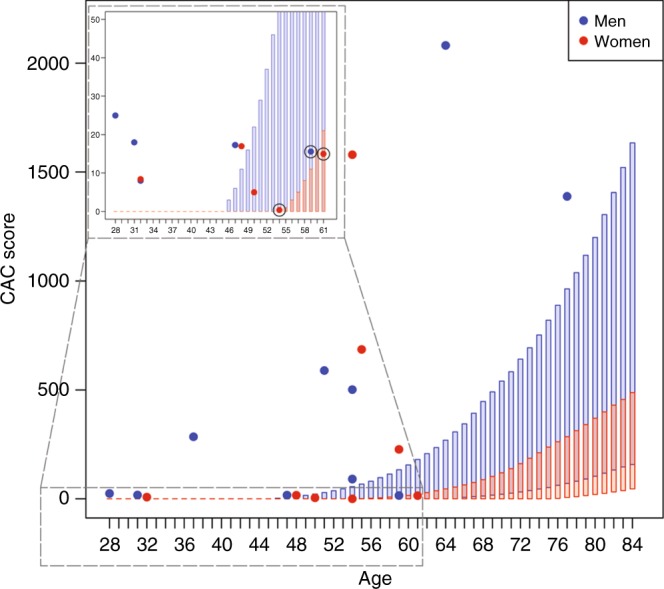
**Subclinical atherosclerotic cardiovascular disease in FH-associated variant carriers.** Coronary artery calcium (CAC) scores of familial hypercholesterolemia (FH)-associated variant carriers with subclinical disease and CAC >0. (*n* = 19) (filled colored circles) in comparison with the distribution of CAC scores in the Multi-Ethnic Study of Atherosclerosis (MESA) subcohort of Caucasian ethnicity and without symptomatic clinical atherosclerotic cardiovascular disease (ASCVD) and treated diabetes. The rectangles represent the expected CAC score distribution between the 25th and 75th percentile in the MESA subcohort for every age and for men (blue) and women (red) separately, with age on the *x*-axis and CAC score on the *y*-axis. While 16 individuals were not expected to have subclinical ASCVD, three individuals did (indicated with black circles). However, the 61-year-old female with *LDLR* p.Gly396Ala and the 54-year-old female with *APOB* p.Arg3527Gln displayed plaques in carotid arteries. The 59-year-old male harbored *LDLR* p.Arg115Cys.

Of 20 individuals with subclinical atherosclerosis, 5 had plaques in carotid arteries and 19 CAC >0. Of the latter group, 16 (84%) were not expected to have subclinical atherosclerosis by the CAC score Reference Value model,^23^ illustrating that the increased LDL-C levels due to a genetic defect predisposes to the premature progression of atherosclerosis. Three individuals, however, displayed expected CAC scores: a 59-year-old male with *LDLR* p.Arg115Cys, and 51- and 61-year-old females harboring *APOB* p.Arg3527Gln and *LDLR* p.Gly396Ala, respectively. The latter two had, however, demonstrable plaques in carotid arteries (Fig. 3).

### Clinical management of FH-associated variant carriers

**Fig. 4:**
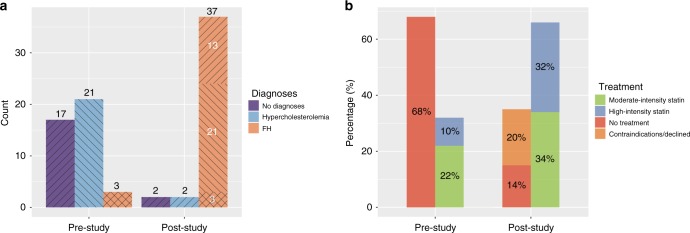
**Clinical management of FH-associated variant carriers.** (**a**) Disease diagnoses in familial hypercholesterolemia (FH)-associated variant carriers before and after the study. After clinical and imaging-based phenotyping, 37 participants were diagnosed with FH: 3 carried FH diagnosis before the study (crossed lines), 21 participants were reclassified from having nonspecific hypercholesterolemia to FH (left-leaning diagonal lines), and 13 were newly diagnosed cases (right-leaning diagonal lines). (**b**) Statin treatment in FH-associated variant carriers before and after the study. At the initial appointment, 13 (32%) participants reported statin use, while 28 (68%) did not. At the end of the study moderate-intensity statin treatment was started for 14 (34%), and up-titrated to or kept on high-intensity statin treatment for 13 (32%) carriers. Statin treatment was not started for 14 (34%) participants, including 8 who either had contraindications or declined.

After clinical and imaging-based phenotyping, 37 individuals (90%) carried a diagnosis for FH. Twenty-one FH-associated variant carriers (51%) were reclassified from having nonspecific hypercholesterolemia to having FH, while 13 (32%) had completely gone unrecognized by the medical system (Fig. 4a). Moderate-intensity statin treatment was started for 14 (34%), statins were up-titrated for 11 (27%), left unchanged for 2 (5%), and not prescribed for 6 (14%). Eight participants (20%) either had contraindications or declined. (Fig. 4b). All individuals were given recommendations for lifestyle modifications.

## Discussion

Recall of probands harboring a high-risk FH-associated genetic variant and their first and second degree family members allowed us to investigate the feasibility and clinical value of the RbG approach for risk estimation and potential risk reduction within a population-based biobank. This strategy led us to ascertain the genetic cause of nonspecific hypercholesterolemia for 51% of the carriers for whom increased LDL-C had clinically been identified, discover 32% who were completely unaware of being at high risk for premature CHD, and allowed us to clinically identify 86% who were targeted for pharmacological intervention.

By screening for FH-associated variants in high-coverage sequencing data (*n* = 4776), we identified 27 probands, of whom 78% participated in the study, and subsequently engaged 76% of the families for clinical evaluation. In total, 41 individuals harbored an FH-associated genetic variant that predisposes to premature CHD. While the increased LDL-C levels had clinically been identified for 51% of the participants prior to the study, only half of them were on statin treatment and none had achieved the guideline-recommended LDL-C levels.^18^ These observations conform with the epidemiological studies denoting substantial underdiagnosis of the disease.^4,24^

The clinical examination of the variant carriers demonstrated insensitivity of current criteria used in FH diagnosis. First, we confirmed that FH-associated variant carriers display a wide spectrum of LDL-C levels as observed previously:^8,9^ 14 (34%) of the 41 variant carriers had statin-adjusted LDL-C levels ≤4.9 mmol/L. Second, the presence of visible accumulations of lipid deposits, which are commonly considered in FH scores (Dutch Lipid Clinic Network,^17^ Simon Broome,^25^ MEDPED^26^), were detected in 5% of the carriers only. And third, we perceived significant heterogeneity in clinical expression, even in individuals carrying the same FH-associated variant. Variability in phenotype and paucity of physical FH features has been highlighted previously,^27,28^ even among homozygous FH cases,^29^ and thus, reinforces the requisite for genetic testing in FH diagnosis.

Via imaging-based phenotyping, we identified premature clinical or subclinical atherosclerosis in 59% of the participants. This corroborates with recent results that the disease is diagnosed late in life and most of the FH cases are characterized with the presence of ASCVD.^30–32^ Participants without demonstrable atherosclerosis (34%) were considerably younger (mean age 36.2 [SD 8.9]) compared with those with subclinical disease (mean age 50.5 [SD.12.8]), and comprise, thus, a target group that should be kept under further clinical surveillance.

This study is among the first that utilizes the genomic and health data contained in a population-based biobank to evaluate the implications of genomics-guided disease management that can be directly translated to the clinic. While the RbG approach has been shown to be an effective tool to systematically investigate tissue-specific and mechanistic associations of single or multiple genetic variants with phenotypes of interest,^1,33,34^ the assessment of clinical impacts of such a study design is thus far lacking. Here we demonstrate that biobank-contained resources hold the potential to determine disease- or phenotype-predisposing genetic variants, identify diagnostic shortcomings in the current medical system, and provide clinically applicable solutions to help fill these gaps. The high call-back rate of study participants and the identification of clinical and genetic unawareness of FH demonstrates the value of such approach and augments the power of biobank-based studies. Implementation of such strategy can ultimately result in more accurate risk estimation of other clinically important phenotypes and guide toward personalized and more appropriate preventative measures. However, we recognize that the value and essence of genetic testing and cascade screening need better communication to the general public and to various levels of health-care providers. Integration of decision-support software into clinical settings can greatly benefit the health-care professionals in terms of interpreting genetic risk.

The study had a number of limitations. First, the number of the identified FH-associated variant carriers was small. Although the yield of probands in the discovery platform reflects the prevalence of FH in Europe,^4,24^ the ascertainment of additional disease-causing genetic variants was limited. While the participation rate of probands and family members demonstrated great interest in the study, the yield of one new case per proband reflects small family sizes in Estonia, illustrates an existing treatment gap in FH cases and highlights the need for increased disease awareness. Cascade screening based on genetic testing has been shown to be incrementally cost-effective for identifying individuals affected by FH, even with similar cascade screening yields,^35–37^ and should, thus, be encouraged. Second, our analysis was limited to single-nucleotide variant discovery. While no copy-number variations >1000 base pairs (bp) were detected in the GS sample set (*n* = 2420), smaller structural rearrangements <1000 bp were identified, but not analyzed. Given the enrichment of *Alu* elements in the *LDLR* gene locus and prior associations of DNA rearrangements with FH,^38,39^ the structural variations can account for a number of FH cases. Lastly, we acknowledge that in silico prediction algorithms for variant pathogenicity prediction do not work perfectly and can indicate false-positive associations. Moreover, given that >1500 variants in the *LDLR* gene have been associated with FH^38^ and discrepancies do exist in NCBI-ClinVar regarding clinical significance of FH-associated variants,^20^ we established a multilevel framework to minimize false-positive variant inclusion. First, we concentrated on rare variants annotated only as loss-of-function or deleterious missense within the FH-associated genes and/or gene domains. Second, we used an endophenotype (LDL-C level) as the intermediate step for filtering out putative false-positive variants that were deemed deleterious based on in silico prediction algorithms. Third, we examined the variant frequencies and prior estimates in databases that outline disease associations or list variants identified in sequencing results of different populations. Finally, variant pathogenicity estimation was leveraged based on deep clinical and imaging-based phenotyping as well as on segregation patterns within families. Our approach conforms with the aims of ClinGen^40^ to improve the understanding of variation within a population, provide expert-reviewed clinical validity, and assess the clinical actionability of variants that can and should be incorporated into clinical setting.

In summary, we established that recalling participants by genotype within a biobank is feasible. Next, we identified the increased need for nationwide testing in Estonia to identify those at increased risk for premature CHD, especially given the actionability of the disease. Finally, we conclude that implementing genomics-guided disease management utilizing the resources contained in a population-based biobank can facilitate clinical management in a more personalized and more effective manner.

## Electronic supplementary material

Supplementary Figure 1

Supplementary Figure 2

Supplementary Table S1

Supplementary Table S2

Supplementary Table S3

Supplementary Table S4

Supplementary Table S5

Supplementary Table S6

Supplementary Information
