# Supplementary figures and images for: Recall by genotype and cascade screening for familial hypercholesterolemia in a population-based biobank from Estonia
Source: Genet Med. 2018 Oct 1;21(5):1173–80. doi: 10.1038/s41436-018-0311-2 (PMC6443485; doi:10.1038/s41436-018-0311-2)

A

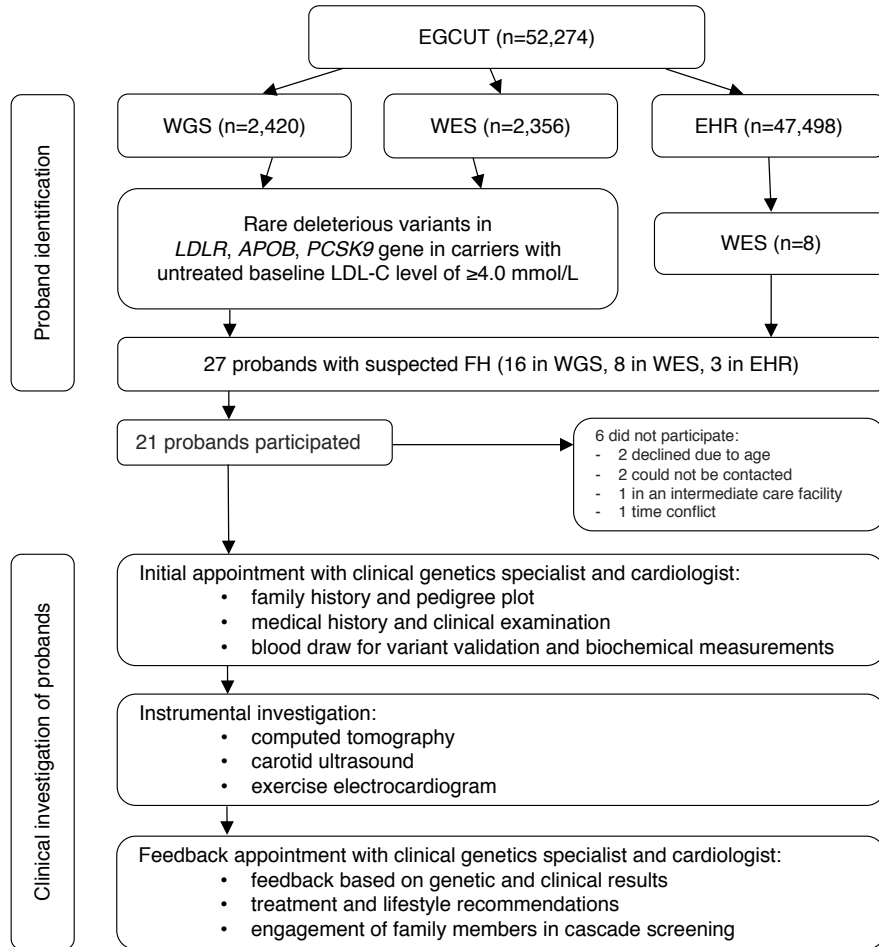

B

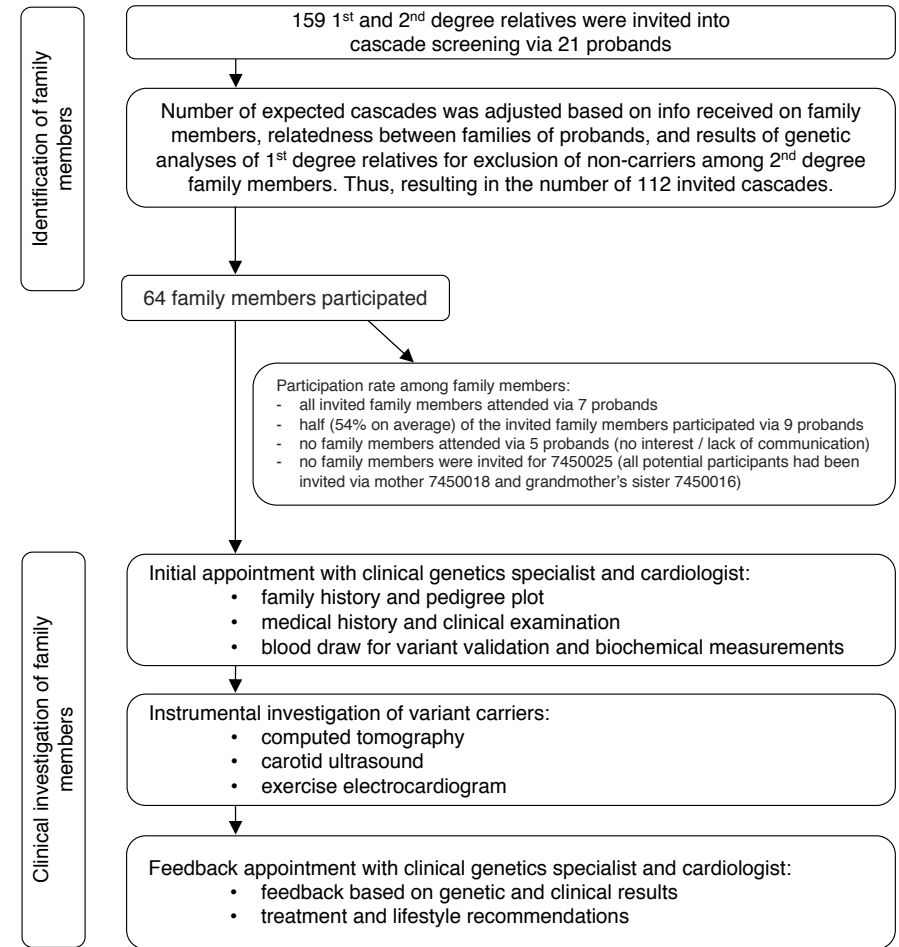

Supplement: Supplementary file 1 — Supplementary Figure 1 [file 41436_2018_311_MOESM1_ESM.pdf]

986 individuals

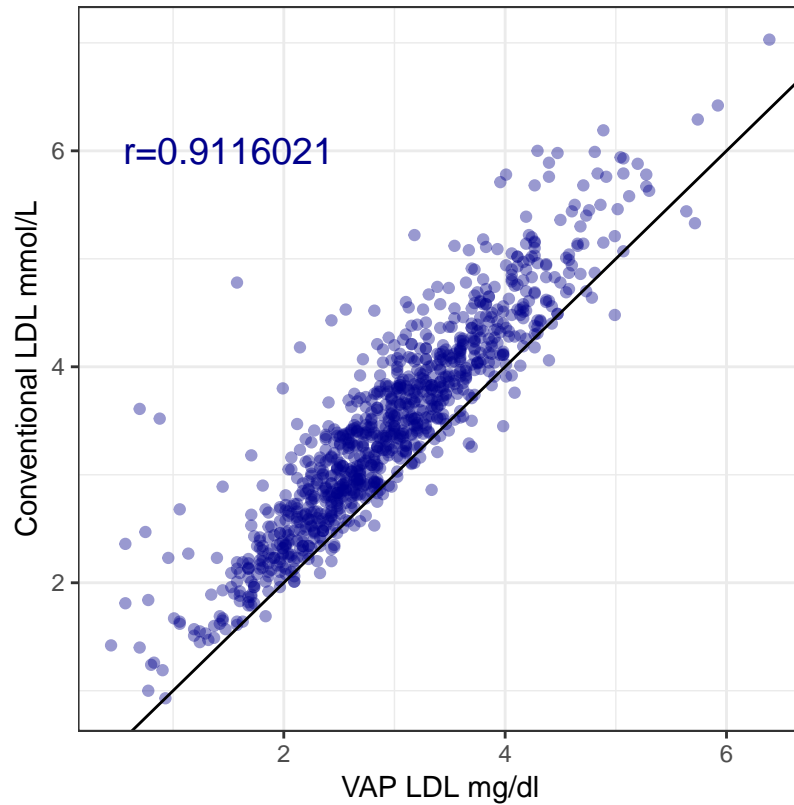

Supplement: Supplementary file 2 — Supplementary Figure 2 [file 41436_2018_311_MOESM2_ESM.pdf]
