# Supplementary material for: Recall by genotype and cascade screening for familial hypercholesterolemia in a population-based biobank from Estonia
Source: Genet Med. 2018 Oct 1;21(5):1173–80. doi: 10.1038/s41436-018-0311-2 (PMC6443485; doi:10.1038/s41436-018-0311-2)
Supplement: Supplementary file 3 — Supplementary Table S1 [file 41436_2018_311_MOESM3_ESM.pdf]

Table S1. Baseline characteristics of high-coverage sequenced individuals at Estonian Genome Center, University of Tartu.

	WGS	WES	Sub-cohort of non-carriers**
N	2,240	2,356	978
Age (years)	46.6 (17.7)	30.6 (7.4)	53.4 (15.8)
Women	1227 (51%)	1017 (43%)	490 (50%)
Blood pressure (mmHg)			
systolic	128.9 (18.5)	118.2 (12.7)	132.7 (19.6)
diastolic	78.9 (11.5)	73.5 (9.0)	83.2 (11.7)
LDL-C (mmol/L)*	3.26 (0.96)	NA	3.56 (0.98)
Diabetes Mellitus	108 (4%)	0 (0%)	51 (5%)
Body mass index	26.9 (5.5)	21.8 (2.8)	27.9 (5.5)
Current smokers	641 (26%)	909 (39%)	179 (18%)
Lipid-lowering medication	152 (7%)	0 (0%)	93 (10%)
Antihypertensive medication	689 (28%)	61 (3%)	369 (38%)

Data are number (%) or mean (SD). *LDL-C levels for the WGS sample set was available for 2,252 individuals; VAP-based LDL-C measurements were scaled to correspond to the conventionally measured LDL-C values using linear regression-based coefficients. LDL-C levels were unavailable for the whole WES sample set and measured for FH-associated variant carriers only. **Sub-cohort of non-carriers for LDL-C level association analysis was formed based on the WGS sample set to exclude false-negatives in the controls and using conventionally measured LDL-C values for LDL-C measurement consistency. WGS - whole-genome sequencing; WES - whole-exome sequencing.
