# Supplementary material for: Recall by genotype and cascade screening for familial hypercholesterolemia in a population-based biobank from Estonia
Source: Genet Med. 2018 Oct 1;21(5):1173–80. doi: 10.1038/s41436-018-0311-2 (PMC6443485; doi:10.1038/s41436-018-0311-2)
Supplement: Supplementary file 4 — Supplementary Table S2 [file 41436_2018_311_MOESM4_ESM.pdf]

Table S2. Dutch Lipid Clinic Network criteria.

	Criteria	Points
Family history	First-degree relative with known premature (men: <55 years; women: <60 years) coronary or vascular disease and/or First-degree relative with known LDL-C above the 95th percentile	1
	First-degree relative with tendinous xanthomata and/or arcus cornealis and/or Children <18 years of age with LDL-C above the 95th percentile	2
Clinical history	Premature (men: <55 years; women: <60 years) coronary artery disease	2
	Premature (men: <55 years; women: <60 years) cerebral or peripheral vascular disease	1
Physical examination	Tendinous xanthomata	6
	Arcus cornealis before age 45 years	4
LDL-C levels	≥ 8.5 mmol/L	8
	6.5–8.4 mmol/L	5
	5.0–6.4 mmol/L	3
	4.0–4.9 mmol/L	1
DNA analysis	Mutation in the *LDLR*, *APOB* or *PCSK9* gene	8
Definite FH		Score >8
Probable FH		Score 6-8
Possible FH		Score 3-5
Unlikely		Score <3

FH - familial hypercholesterolemia; LDL-C - LDL cholesterol
