# Supplementary material for: Recall by genotype and cascade screening for familial hypercholesterolemia in a population-based biobank from Estonia
Source: Genet Med. 2018 Oct 1;21(5):1173–80. doi: 10.1038/s41436-018-0311-2 (PMC6443485; doi:10.1038/s41436-018-0311-2)
Supplement: Supplementary file 5 — Supplementary Table S3 [file 41436_2018_311_MOESM5_ESM.pdf]

Table S3. Identified FH-associated variants.

Gene	Chr	GRCh37/hg19 position	rs-number	Variant info (exon; codon; protein)	AC_EST*	Polyphen2 HdivPred	SIFT	Condel	PhyloP	CADD	MutationTaster	Clinvar	gnomAD_total**	EXAC***	SISU****	UCL LDLR gene variant database; ACSG class	HGMD	Variant categorication after study based on ACMG criteria
APOB	2	21229160	rs5742904	26/29; c.10580G>A; p.Arg3527Gln	AC=11	probably damaging	NA	Neutral	4.569	29.4	disease causing	Conflicting interpretations of pathogenicity	AC=79	AC=28	AC=0	NA	1	likely pathogenic
LDLR	19	11215925	rs774723292	4/18; c.343C>T; p.Arg115Cys	AC=1	probably damaging	Deleterious	Deleterious	3.927	33	disease causing	Conflicting interpretations of pathogenicity	AC=7	AC=4	AC=0	ACGS=4	1	VUS
LDLR	19	11216225	rs764042910	4/18; c.643C>T; p.Arg215Cys	AC=1	probably damaging	Deleterious	Deleterious	0.619	28.1	disease causing	Pathogenic	AC=15	AC=2	AC=0	NA; c.643C>A, p.Arg215Ser	0	likely pathogenic
LDLR	19	11217295	NA	5/18; c.749A>G; p.His250Arg	AC=2	probably damaging	Deleterious	Deleterious	2.036	22.9	disease causing	NA	AC=1	AC=0	AC=0	NA; c.750T>C; p.His250=	0	VUS
LDLR	19	11221373	rs761954844	7/18; c.986G>A; p.Cys329Tyr	AC=5	probably damaging	Deleterious	Deleterious	5.767	25.7	disease causing	Conflicting interpretations of pathogenicity	AC=7	AC=0	AC=0	ACGS=4	1	likely pathogenic
LDLR	19	11223954	rs766474188	9/18; c.1187G>C; p.Gly396Ala	AC=1	probably damaging	Deleterious	Deleterious	2.462	23.2	disease causing	NA	AC=3	AC=4	AC=1	NA; c.1186G>A, p.Gly396Ser	0	likely pathogenic
LDLR	19	11223969	rs121908038	9/18; c.1202T>A; p.Leu401His	AC=2	probably damaging	Deleterious	Deleterious	4.546	25.5	disease causing	Pathogenic/Likely pathogenic	AC=0	AC=0	AC=0	ACGS=4	1	likely pathogenic
LDLR	19	11224058	rs28942079	9/18; c.1291G>T; p.Ala431Ser	AC=1	benign	Deleterious	Deleterious	5.506	30	disease causing	NA; p.Ala431Thr, p.Ala431Pro	AC=1	AC=0	AC=0	NA; c.1291G>A, p.Ala431Thr	0	likely pathogenic
LDLR	19	11224074	rs779732323	9/18; c.1307T>C; p.Val436Ala	AC=1	benign	Deleterious	Neutral	4.546	26.5	disease causing	Conflicting interpretations of pathogenicity	AC=5	AC=1	AC=0	ACGS=3	1	VUS
LDLR	19	11230820	rs754536745	13/18; c.1898G>A; p.Arg633His	AC=1	probably damaging	Deleterious	Deleterious	3.138	27.5	disease causing	Conflicting interpretations of pathogenicity	AC=6	AC=2	AC=0	ACGS=4; c.1897C>T, p.Arg633Cys	1	likely pathogenic
PCSK9	1	55523076	rs148562777	7/12; c.1069C>T; p.Arg357Cys	AC=1	probably damaging	Deleterious	Deleterious	1.589	34	disease causing	NA; p.Arg357His	AC=43	AC=18	AC=0	NA	0	likely pathogenic

of unknown signifcance

*Query based on 2,240 whole-genome sequences and 2,356 whole-exome sequences.

**Query based on 123,136 exome sequences and 15,496 whole-genome sequences at http://gnomad.broadinstitute.org/

***Query based on 60,706 exome sequences at http://exac.broadinstitute.org/

****Query based on 10,490 exome sequences at http://www.sisuproject.fi/
