# Supplementary material for: Recall by genotype and cascade screening for familial hypercholesterolemia in a population-based biobank from Estonia
Source: Genet Med. 2018 Oct 1;21(5):1173–80. doi: 10.1038/s41436-018-0311-2 (PMC6443485; doi:10.1038/s41436-018-0311-2)
Supplement: Supplementary file 6 — Supplementary Table S4 [file 41436_2018_311_MOESM6_ESM.pdf]

Table S4. Baseline characteristics of probands.

IID	Gene	Variant info	BirthYear	Sex	HC in EHR	CHD in EHR	Statin prescription in EHR	Baseline statin use	VAP TC mg/dl	VAP LDLR mg/dl	VAP LDLR transformed mmol/L	C TC mmol/L	C LDL mmol/L	Baseline LDL-C mmol/L	Baseline LDL-C mmol/L statin-adjusted	Participation
7450001	*APOB*	c.10580G>A; p.Arg3527Gln	1983	Male	E78.0	0	1	0	331	212	5.9335	NA	NA	5.93	5.93	1
7450002	*APOB*	c.10580G>A; p.Arg3527Gln	1961	Male	E78.0	0	1	0	276	175	4.9933	NA	NA	4.99	4.99	1
7450004	*APOB*	c.10580G>A; p.Arg3527Gln	1936	Male	E78.0	I21 I25.1 I25.9 Z95.1	1	0	293	196	5.5269	7.4	5.07	5.07	5.07	1
7450009	*APOB*	c.10580G>A; p.Arg3527Gln	1962	Male	E78.0	0	1	0	NA	NA	NA	7.6	4.8	4.8	4.8	1
7450015	*APOB*	c.10580G>A; p.Arg3527Gln	1969	Female	E78.5	0	1	0	NA	NA	NA	3.9	2.58	2.58	2.58	1
7450016	*APOB*	c.10580G>A; p.Arg3527Gln	1937	Female	E78.0	0	1	1	394	23	6.5180	NA	NA	6.52	9.31	1
7450018	*APOB*	c.10580G>A; p.Arg3527Gln	1961	Female	E78.0_2 (FH)	0	0	0	NA	NA	NA	10.0	7.55	7.55	7.55	1
7450023	*APOB*	c.10580G>A; p.Arg3527Gln	1975	Female	E78	0	0	0	NA	NA	NA	8.0	6.05	6.05	6.05	1
7450025	*APOB*	c.10580G>A; p.Arg3527Gln	1984	Female	E78.2	0	1	1	414	247	6.8229	9.3	7.03	7.03	10.04	1
7450028	*APOB*	c.10580G>A; p.Arg3527Gln	1969	Male	E78.0	0	0	0	NA	NA	NA	7.5	5.90	5.9	5.9	1
7484000	*APOB*	c.10580G>A; p.Arg3527Gln	1969	Female	0	0	0	0	NA	NA	NA	8.7	6.57	6.57	6.57	0
7450013	*LDLR*	c.343C>T; p.Arg115Cys	1983	Female	0	0	0	0	NA	NA	NA	4.6	2.89	2.89	2.89	1
7450026	*LDLR*	c.643C>T; p.Arg215Cys	1952	Male	E78	0	1	0	314	162	4.6629	NA	NA	4.66	4.66	1
7450032	*LDLR*	c.749A>G; p.His250Arg	1975	Male	0	0	0	0	NA	NA	NA	5.5	3.67	3.67	3.67	1
7482000	*LDLR*	c.749A>G; p.His250Arg	1933	Female	E78.0	I24.9 I20 I25	1	1	219	112	3.3924	5.1	3.23	3.23	4.61	0
7450003	*LDLR*	c.986G>A; p.Cys329Tyr	1967	Female	E78.0_2 (FH)	0	1	1	NA	NA	NA	5.7	4.21	4.21	6.01	1
7450006	*LDLR*	c.986G>A; p.Cys329Tyr	1988	Male	E78.0	0	1	1	319	211	5.9081	NA	NA	5.91	8.44	1
7450019	*LDLR*	c.986G>A; p.Cys329Tyr	1962	Female	E78.0_2 (FH)	0	1	1	NA	NA	NA	7.58	3.3	3.3	4.71	1
7481200	*LDLR*	c.986G>A; p.Cys329Tyr	1967	Female	E78.0	0	1	0	NA	NA	NA	9.2	7.26	7.26	7.26	0
7483000	*LDLR*	c.986G>A; p.Cys329Tyr	1975	Female	E78.0	0	1	0	278	189	NA	6.4	5.15	5.15	5.15	0
7450020	*LDLR*	c.1187G>C; p.Gly396Ala	1977	Male	0	0	0	0	225	134	3.9514	5.8	3.74	3.74	3.74	1
7450029	*LDLR*	c.1202T>A; p.Leu401His	1985	Male	0	0	0	0	NA	NA	NA	6.5	5.22	5.22	5.22	1
7481300	*LDLR*	c.1202T>A; p.Leu401His	1978	Male	0	0	0	0	NA	NA	NA	7.6	6.10	6.1	6.1	0
7450007	*LDLR*	c.1291G>T; p.Ala431Ser	1979	Male	E78.0	0	1	0	320	190	5.3744	8.3	5.76	5.76	5.76	1
7481000	*LDLR*	c.1307T>C; p.Val436Ala	1934	Female	E78.0	I20.8 I21 I25.0 Z95.5	1	0	325	182	5.1712	7.6	5.14	5.14	5.14	0
7450014	*LDLR*	c.1898G>A; p.Arg633His	1942	Male	E78	I20.8 I25.8 Z95.1	1	1	205	92	2.8841	5.7	3.35	3.35	4.79	1
7450022	*PCSK9*	c.1069C>T; p.Arg357Cys	1984	Female	0	0	0	0	325	178	5.0695	NA	NA	5.07	5.07	1

HC - hypercholesterolemia (reported as ICD-10 code); ASCVD - atherosclerotic cardiovascular disease (reported as ICD-10 code); EHR - electronic health records; VAP - vertical auto profile for lipid measurements; C - conventional
