# Supplementary material for: Recall by genotype and cascade screening for familial hypercholesterolemia in a population-based biobank from Estonia
Source: Genet Med. 2018 Oct 1;21(5):1173–80. doi: 10.1038/s41436-018-0311-2 (PMC6443485; doi:10.1038/s41436-018-0311-2)
Supplement: Supplementary file 7 — Supplementary Table S5 [file 41436_2018_311_MOESM7_ESM.pdf]

Table S5. Characteristics of all study participants.

ID	Proband/cascade	Gene	Variant info	Carrier status	Cascade participation	Modified cascade participation	Relatedness	Age	Sex	Pre-study diagnosis	Family history of CHD*	Pre-study statin prescription	Pre-study statin use	TC mmol/L	TC statin adjusted mmol/L	LDL mmol/L	LDL statin adjusted mmol/L	HDL mmol/L	TG mmol/L
7450001	1	*APOB*	c.10580G>A; p.Arg3527Gln	1	1	1	NA	32	Male	hypercholesterolemia at 22yo	no	1	0	7.4	7.4	5.87	5.87	1.61	0.76
7450101	2			0			mother	56	Female	hypercholesterolemia at 40yo	NA	1	atorvastatin 20mg	3.8	4.75	2.28	3.26	1.17	1.21
7450002	1	*APOB*	c.10580G>A; p.Arg3527Gln	1	3	3	NA	54	Male	hypercholesterolemia at 48yo	no	1	rosuvastatin 10mg	5.3	7.57	3.81	5.44	1.07	1.55
7450102	2			1			son	32	Male	0	no	0	0	8.6	8.6	7.03	7.03	1.11	2.48
7450003	1	*LDLR*	c.986G>A; p.Cys329Tyr	1	12	9	NA	48	Female	FH suspected at 32yo	yes	1	rosuvastatin 10mg	5.4	7.7	3.12	4.46	2.02	0.83
7450103	2			1			brother	51	Male	increased TC level at 42yo	yes	1	rosuvastatin 20mg; ezetimibe 10mg	6.3	7.88	4.88	6.97	1.27	1.11
7450203	2			0			father	73	Male	CHD; stenting	NA	1	atorvastatin 20mg	3.3	4.13	1.49	2.13	1.60	0.82
7450303	2			0			son	18	Male	0	NA	0	0	3.4	3.4	2.12	2.12	1.15	1.04
7450403	2			0			son	23	Male	0	NA	0	0	4.3	4.3	2.96	2.96	1.17	1.10
7450503	2			0			father's sister	71	Female	CHD at 69yo	NA	1	atorvastatin 10mg	4.6	5.75	2.59	3.70	1.84	1.14
7450603	2			0			mother's sister	71	Female	0	NA	1	simvastatin 20mg	6.4	8.00	4.24	6.06	1.64	1.66
7450703	2			1			daughter	26	Female	hypercholesterolemia diagnosed at adolescence	no	0	0	6.8	6.8	5.29	5.29	1.63	0.81
7450803	2			0			brother	43	Male	0	NA	0	0	5.4	5.4	4.08	4.08	1.39	1.09
7450004	1	*APOB*	c.10580G>A; p.Arg3527Gln	1	8	5	NA	81	Male	MI at 46yo; recurrent MI at 75yo; hypercholesterolemia at 72yo	no	1	0	8.2	8.2	5.97	5.97	2.05	0.62
7450104	2			1			daughter	51	Female	hypercholesterolemia at 36yo	yes	1	atorvastatin 40mg	5.2	6.5	3.33	4.76	2.01	0.44
7450204	2			1			sister	82	Female	stroke at 70yo; increaded TC level	no	1	simvastatin 40mg	6.4	8	4.84	6.90	1.44	1.37
7450304	2			0			daughter	50	Female	0	NA	0	0	6.0	6.0	3.70	3.70	2.35	0.80
7450404	2			0			daughter's son	24	Male	0	NA	0	0	5.9	5.9	4.20	4.20	1.92	0.68
7450504	2			1			daughter's son	32	Male	0	no	0	0	7.8	7.8	6.39	6.39	1.36	1.89
7450006	1	*LDLR*	c.986G>A; p.Cys329Tyr	1	4	1	NA	28	Male	hypercholesterolemia at 14yo	no	1	0	10.9	10.9	9.21	9.21	0.92	1.78
7450106	2			0			mother	48	Female	0	NA	0	0	5.8	5.8	3.85	3.85	1.32	2.52
7450007	1	*LDLR*	c.1291G>T; p.Ala431Ser	1	12	10	NA	37	Male	hypercholesterolemia at 32yo	no	1	atorvastatin 10mg	6	7.5	4.39	6.27	1.38	1.05
7450107	2			0			father's sister	57	Female	mild hypercholesterolemia at 56yo	NA	0	0	6.4	6.4	4.43	4.43	2.13	0.68
7450207	2			0			father	66	Male	CHD at <50yo; CABG	NA	0	0	6.8	6.8	5.16	5.16	1.05	1.83
7450307	2			1			mother	59	Female	hypercholesterolemia at 56yo	no	0	0	8.0	8.0	5.56	5.56	2.83	0.60
7450009	1	*APOB*	c.10580G>A; p.Arg3527Gln	1	6	6	NA	54	Male	hypercholesterolemia at 45yo	no	1	0	8.9	8.9	6.31	6.31	3.63	0.58
7450109	2			0			daughter	31	Female	0	NA	0	0	5.4	5.4	3.60	3.60	2.01	0.75
7450209	2			0			half-brother	66	Male	0	NA	0	0	6.2	6.2	4.96	4.96	1.81	0.61
7450309	2			1			son	25	Male	0	no	0	0	5.4	5.4	3.74	3.74	1.72	0.55
7450409	2			0			son	30	Male	0	NA	0	0	4.5	4.5	2.55	2.55	2.08	0.58
7450509	2			0			brother's daughter	27	Female	0	NA	0	0	5.0	5.0	2.60	2.60	2.28	0.91
7450609	2			0			brother's son	29	Male	0	NA	0	0	4.4	4.4	2.76	2.76	1.52	0.75
7450013	1	*LDLR*	c.343C>T; p.Arg115Cys	1	5	5	NA	33	Female	0	no	0	0	5.5	5.5	3.72	3.72	1.89	0.60
7450113	2			1			brother	26	Male	0	no	0	0	4.1	4.1	3.22	3.22	1.20	0.61
7450213	2			0			mother's brother	62	Male	0	NA	0	0	6.4	6.4	4.52	4.52	1.46	1.30
7450313	2			0			mother's twin brother	59	Male	0	NA	0	0	5.8	5.8	3.62	3.62	1.49	2.41
7450413	2			1			father	59	Male	0	no	0	0	6.8	6.8	5.22	5.22	1.30	1.55
7450513	2			0			mother	59	Female	0	NA	0	0	6.7	6.7	4.60	4.60	1.52	2.12
7450014	1	*LDLR*	c.1898G>A; p.Arg633His	1	15	10	NA	74	Male	hypercholesterolemia at 61yo; CHD; stenting	no	1	rosuvastatin 10mg	4.2	5.25	2.72	3.89	1.13	1.49
7450114	2			0			sister's daughter	36	Female	0	NA	0	0	4.6	4.6	3.23	3.23	1.69	0.56
7450214	2			0			sister	59	Female	hypercholesterolemia at 35yo	NA	0	phenofibrate 200mg	4.5	4.5	2.70	2.70	1.92	0.77
7450314	2			0			sister's daughter	37	Female	0	NA	0	0	5.2	5.2	3.71	3.71	1.44	0.88
7450414	2			0			sister	76	Female	angina pectoris at 60yo	NA	1	rosuvastatin 10mg	3.9	4.875	1.80	2.57	2.27	0.54
7450514	2			0			sister's daughter	33	Female	0	NA	0	0	4.9	4.9	3.58	3.58	1.32	0.62
7450614	2			1			son	48	Male	0	no	0	0	5.8	5.8	4.08	4.08	2.07	0.57
7450015	1	*APOB*	c.10580G>A; p.Arg3527Gln	1	7	7	NA	47	Female	hypercholesterolemia at 36yo	no	1	rosuvastatin 10mg	4.7	6.7	2.82	4.03	1.98	0.54
7450115	2			1			sister's daughter	27	Female	0	no	0	0	6.0	6.0	4.87	4.87	1.27	0.52
7450215	2			1			daughter	50	Female	hypercholesterolemia and hypertension at 40yo	no	1	rosuvastatin 5mg	6.0	7.5	4.33	6.19	1.38	1.26
7450315	2			1			sister	25	Female	0	no	0	0	6.3	6.3	4.27	4.27	2.18	0.99
7450016	1	*APOB*	c.10580G>A; p.Arg3527Gln	1	17	7	NA	78	Female	hypercholesterolemia at 68yo (arcus cornealis)	yes	1	rosuvastatin 20mg	8.0	11.43	6.37	9.1	1.78	1.15
7450116	2			1			brother	77	Male	hypercholesterolemia (arcus cornealis); hypertension at 59yo	yes	1	rosuvastatin 10mg	5.3	6.625	4.19	5.99	1.22	1.07
7450216	2			1			daughter	55	Female	0	no	0	0	13.6	13.6	11.56	11.56	1.59	2.09
7450316	2			0			brother's son	28	Male	0	NA	0	0	4.4	4.4	2.98	2.98	1.39	0.67
7450416	2			0			brother's daughter	30	Female	0	NA	0	0	6.4	6.4	4.90	4.90	1.2	1.6
7450516	2			0			brother's son	44	Male	0	NA	0	0	5.5	5.5	3.50	3.50	1.7	1.0
7450018	1	*APOB*	c.10580G>A; p.Arg3527Gln	1	12	5	NA	54	Female	FH supsected at 45yo	no	0	0	11.0	11.0	9.08	9.08	1.81	1.37
7450118	2			0			brother's daughter	34	Female	0	NA	0	0	4.6	4.6	3.33	3.33	1.32	0.73
7450218	2			0			daughter	31	Female	0	NA	0	0	6.0	6.0	4.03	4.03	2.18	1.08
7450019	1	*LDLR*	c.986G>A; p.Cys329Tyr	1	8	7	NA	54	Female	hypercholesterolemia at 35yo	yes	1	rosuvastatin 20mg	7.9	11.29	4.03	5.76	4.38	0.56
7450119	2			0			son	31	Male	0	NA	0	0	5.6	5.6	3.27	3.27	2.21	1.46
7450219	2			0			father's sister	72	Female	0	NA	1	atorvastatin 20mg	5.1	6.38	3.06	4.37	1.29	1.96
7450319	2			0			father's sister	67	Female	stroke at 60yo	NA	1	rosuvastatin 10mg	4.7	5.91	2.51	3.59	2.04	1.36
7450419	2			0			brother's son	35	Male	0	NA	0	0	5.0	5.03	3.36	3.36	1.64	0.91
7450519	2			0			brother's son	26	Male	0	NA	0	0	3.9	3.87	2.09	2.09	1.82	0.69
7450619	2			0			brother's son	28	Male	0	NA	0	0	4.3	4.3	2.70	2.70	1.8	0.6
7450719	2			0			son	59	Male	MI at 48yo	NA	1	simvastatin 40mg	4.8	6.00	2.92	4.17	1.43	1.71
7450020	1	*LDLR*	c.1187G>C; p.Gly396Ala	1	8	7	NA	39	Male	0	no	0	0	6.1	6.1	4.41	4.41	0.95	2.64
7450120	2			0			half-sister	32	Female	0	NA	0	0	5.8	5.8	3.48	3.48	2.44	0.64
7450220	2			1			mother's sister	57	Female	MI at 53yo; angina pectoris 52yo; hypercholesterolemia suspected	no	1	0	8.2	8.2	5.77	5.77	1.37	4.00
7450320	2			0			mother's sister	50	Female	0	NA	0	0	6.0	6.02	3.96	3.96	1.10	3.63
7450420	2			1			mother	61	Female	0	no	0	0	5.8	5.8	4.20	4.20	1.2	1.7
7450520	2			0			mother's brother	68	Male	0	NA	0	0	6.3	6.3	4.55	4.55	1.64	1.31
7450022	1	*PCSK9*	c.1069C>T; p.Arg357Cys	1	8	4	NA	32	Female	0	no	0	0	7.5	7.5	5.06	5.06	1.87	1.94
7450122	2			0			mother	67	Female	0	NA	0	0	7.2	7.2	5.65	5.65	1.13	1.99
7450222	2			0			brother	37	Male	0	NA	0	0	5.4	5.4	3.88	3.88	1.31	0.66
7450322	2			0			half-brother	53	Male	0	NA	0	0	5.9	5.9	4.52	4.52	1.13	1.51
7450422	2			0			brother	39	Male	0	NA	0	0	4.9	4.9	3.35	3.35	1.60	0.52
7450023	1	*APOB*	c.10580G>A; p.Arg3527Gln	1	4	3	NA	41	Female	hypercholesterolemia at 32yo	no	0	0	7.2	7.2	5.22	5.22	2.12	0.64
7450025	1	*APOB*	c.10580G>A; p.Arg3527Gln	1	0	0	NA	32	Female	FH suspected at 23yo	no	1	0	12.7	12.7	10.41	10.41	2.45	0.96
7450026	1	*LDLR*	c.643 C>T; p.Arg215Cys	1	8	7	NA	64	Male	hypercholesterolemia at 54yo	no	1	rosuvastatin 10mg	6.4	8	3.82	5.46	1.85	2.97
7450126	2			1			son	39	Male	0	no	0	0	6.1	6.1	4.00	4.00	1.3	2.40
7450226	2			1			sister	63	Female	0	no	0	0	7.7	7.7	5.50	5.50	1.8	2.1
7450028	1	*APOB*	c.10580G>A; p.Arg3527Gln	1	10	5	NA	47	Male	hypercholesterolemia at 39yo	no	0	Reg'Activ natural supplement	7.6	7.6	5.54	5.54	1.70	1.13
7450029	1	*LDLR*	c.1202T>A; p.Leu401His	1	9	8	NA	31	Male	0	no	0	0	7.9	7.9	6.49	6.49	1.09	0.61
7450032	1	*LDLR*	c.749A>G; p.His250Arg	1	2	2	NA	42	Male	0	no	0	0	5.4	5.4	3.92	3.92	1.42	1.10

*First-degree relative with known premature (men: <55 years; women: <60 years) coronary or vascular disease. CAC - coronary artery calcium score; ECG - exercise electrocardiogram; IMT - intima-media thickness; CHD - coronary heart disease; MI - myocardial infarction; CABG - coronary artery bypass grafting; TC - total cholesterol, TG - triglycerides; DLCN FH score - Dutch Lipid Clinic Network Familial Hypercholest

ID	CAC (Agatston score)	exercise ECG	carotid ultrasound: average IMT (mm); presence of plaque	Imaging results	DLCN FH score	Post-study statin	Final diagnosis
7450001	LAD 8	no ischemia	0.8	sub-clinical ASCVD	11	atorvastatin 10mg	FH
7450101	NA	NA	NA	NA	NA	NA	NA
7450002	LCX 8, RCA 83	ischemia suspected	NA	sub-clinical ASCVD	13	rosuvastatin 10mg	FH
7450102	0	no ischemia	0.9	no sub-clinical ASCVD	14	not prescribed due to liver problems	FH
7450003	LAD 13, LCX 4	no ischemia	0.72	sub-clinical ASCVD	15	rosuvastatin 20mg	FH
7450103	589 (RCA, LAD, LCX)	no ischemia	1.7	sub-clinical ASCVD	9	rosuvastatin 40mg; ezetimibe 10mg	FH
7450203	NA	NA	NA	NA	NA	NA	NA
7450303	NA	NA	NA	NA	NA	NA	NA
7450403	NA	NA	NA	NA	NA	NA	NA
7450503	NA	NA	NA	NA	NA	NA	NA
7450603	NA	NA	NA	NA	NA	NA	NA
7450703	NA (declined)	no ischemia	0.47	declined	12	not prescribed, pregnant	FH
7450803	NA	NA	NA	NA	NA	NA	NA
7450004	NA (disease manifested)	NA (disease manifested)	NA (disease manifested)	prevalent ASCVD	13	rosuvastatin 10mg	FH
7450104	0	no ischemia	0.7	no sub-clinical ASCVD	9	atorvastatin 60mg	FH
7450204	NA (disease manifested)	NA (disease manifested)	NA (disease manifested)	prevalent ASCVD	14	atorvastatin 20mg	FH
7450304	NA	NA	NA	NA	NA	NA	NA
7450404	NA	NA	NA	NA	NA	NA	NA
7450504	0	no ischemia	0.6	no sub-clinical ASCVD	12	atorvastatin 20mg	FH
7450006	25 LAD	no ischemia	0.8	sub-clinical ASCVD	16	atorvastatin 40mg	FH
7450106	NA	NA	NA	NA	NA	NA	NA
7450007	LAD 41, LCX 10, RCA 234	no ischemia	0.7	sub-clinical ASCVD	3	atorvastatin 20mg	FH
7450107	NA	NA	NA	NA	NA	NA	NA
7450207	NA	NA	NA	NA	NA	NA	NA
7450307	LM 14, LAD 83, LCX 5, RCA 125	no ischemia	1.2	sub-clinical ASCVD	4	rosuvastatin 10mg	FH
7450009	LAD 120, LCX 157, RCA 225	no ischemia	1; plaque in carotid artery	sub-clinical ASCVD	11	atorvastatin 20mg	FH
7450109	NA	NA	NA	NA	NA	NA	NA
7450209	NA	NA	NA	NA	NA	NA	NA
7450309	0	no ischemia	0.58	no sub-clinical ASCVD	9	not prescribed	FH
7450409	NA	NA	NA	NA	NA	NA	NA
7450509	NA	NA	NA	NA	NA	NA	NA
7450609	NA	NA	NA	NA	NA	NA	NA
7450013	0	no ischemia	0.5	no sub-clinical ASCVD	0	not prescribed	hypercholesterolemia
7450113	0	no ischemia	0.55	no sub-clinical ASCVD	0	not prescribed	NA
7450213	NA	NA	NA	NA	NA	NA	NA
7450313	NA	NA	NA	NA	NA	NA	NA
7450413	15.6	no ischemia	1.1	sub-clinical ASCVD	4	not prescribed	NA
7450513	NA	NA	NA	NA	NA	NA	NA
7450014	NA (disease manifested)	NA (disease manifested)	NA (disease manifested)	prevalent ASCVD	13	rosuvastatin 20mg	FH
7450114	NA	NA	NA	NA	NA	NA	NA
7450214	NA	NA	NA	NA	NA	NA	NA
7450314	NA	NA	NA	NA	NA	NA	NA
7450414	NA	NA	NA	NA	NA	NA	NA
7450514	NA	NA	NA	NA	NA	NA	NA
7450614	0	no ischemia	1	no sub-clinical ASCVD	10	rosuvastatin 10mg	FH
7450015	0	no ischemia	0.9	no sub-clinical ASCVD	14	rosuvastatin 10mg	FH
7450115	0	no ischemia	0.58	no sub-clinical ASCVD	9	not prescribed, contraindications	FH
7450215	LAD 5	no ischemia	0.57	sub-clinical ASCVD	14	rosuvastatin 20mg	FH
7450315	0	no ischemia	0.6	no sub-clinical ASCVD	9	not prescribed, contraindications	FH
7450016	NA (declined)	no ischemia	1.2	declined	17	rosuvastatin 40mg; ezetimibe 10mg	FH
7450116	LMA 107, LAD 957, LCX 286, RCA 39	no ischemia	1.18; plaque in carotid artery	sub-clinical ASCVD	14	rosuvastatin 20mg	FH
7450216	686	no ischemia	0.7	sub-clinical ASCVD	17	rosuvastatin 20mg	FH
7450316	NA	NA	NA	NA	NA	NA	NA
7450416	NA	NA	NA	NA	NA	NA	NA
7450516	NA	NA	NA	NA	NA	NA	NA
7450018	0.4	no ischemia	1.3; plaque in carotid artery	sub-clinical ASCVD	17	rosuvastatin 20mg	FH
7450118	NA	NA	NA	NA	NA	NA	NA
7450218	NA	NA	NA	NA	NA	NA	NA
7450019	1580	no ischemia	0.95	sub-clinical ASCVD	9	rosuvastatin 40mg	FH
7450119	NA	NA	NA	NA	NA	NA	NA
7450219	NA	NA	NA	NA	NA	NA	NA
7450319	NA	NA	NA	NA	NA	NA	NA
7450419	NA	NA	NA	NA	NA	NA	NA
7450519	NA	NA	NA	NA	NA	NA	NA
7450619	NA	NA	NA	NA	NA	NA	NA
7450719	NA	NA	NA	NA	NA	NA	NA
7450020	0	no ischemia	1	no sub-clinical ASCVD	1	not prescribed	FH
7450120	NA	NA	NA	NA	NA	NA	NA
7450220	NA (disease manifested)	NA (disease manifested)	NA (disease manifested)	prevalent ASCVD	5	not prescribed due to liver problems	FH
7450320	NA	NA	NA	NA	NA	NA	NA
7450420	LAD 8, LCX 2, RCA 5	hypertension suspected	1.12; plaque in carotid artery	sub-clinical ASCVD	2	atorvastatin 20mg	FH
7450520	NA	NA	NA	NA	NA	NA	NA
7450022	8.4	no ischemia	0.7	sub-clinical ASCVD	4	not prescribed, declined	FH
7450122	NA	NA	NA	NA	5	NA	NA
7450222	NA	NA	NA	NA	NA	NA	NA
7450322	NA	NA	NA	NA	NA	NA	NA
7450422	NA	NA	NA	NA	NA	NA	NA
7450023	0	no ischemia	0.9	no sub-clinical ASCVD	12	atorvastatin 20mg	FH
7450025	NA (breastfeeding)	no ischemia	0.9	declined	17	not prescribed, breastfeeding	FH
7450026	2082	no ischemia	1.2	sub-clinical ASCVD	9	rosuvastatin 20mg	FH
7450126	0	no ischemia	0.75	no sub-clinical ASCVD	10	not prescribed, declined	FH
7450226	0	no ischemia	0.77; plaque in carotid artery	sub-clinical ASCVD	12	rosuvastatin 10mg	FH
7450028	17.3	no ischemia	0.8	sub-clinical ASCVD	14	atorvastatin 40mg	FH
7450029	LAD 17, RCA 1	no ischemia	0.8	sub-clinical ASCVD	12	atorvastatin 10mg	FH
7450032	0	no ischemia	0.72	no sub-clinical ASCVD	0	not prescribed	hypercholesterolemia

terolemia score
