# Supplementary material for: Recall by genotype and cascade screening for familial hypercholesterolemia in a population-based biobank from Estonia
Source: Genet Med. 2018 Oct 1;21(5):1173–80. doi: 10.1038/s41436-018-0311-2 (PMC6443485; doi:10.1038/s41436-018-0311-2)
Supplement: Supplementary file 8 — Supplementary Table S6 [file 41436_2018_311_MOESM8_ESM.pdf]

Table S6. Transcript info of FH-associated genes.

Gene	Transcript ID	RefSeq Transcript ID
APOB	ENST00000233242	NM_000527
LDLR	ENST00000558518	NM_174936
PCSK9	ENST00000302118	NM_000384
