# Supplementary material for: Recall by genotype and cascade screening for familial hypercholesterolemia in a population-based biobank from Estonia
Source: Genet Med. 2018 Oct 1;21(5):1173–80. doi: 10.1038/s41436-018-0311-2 (PMC6443485; doi:10.1038/s41436-018-0311-2)
Supplement: Supplementary file 9 — Supplementary Information [file 41436_2018_311_MOESM9_ESM.docx]

SUPPLEMENTARY MATERIALS AND METHODS

**WGS and WES sequencing and variant calling**

WGS followed a PCR-free sample preparation and sequenced on the Illumina HiSeq X Ten using 150 bp paired-end reads with mean coverage of 30×. WES samples were sequenced using the Agilent SureSelect Human All Exon V5+UTRs Kit according to the manufacturer’s recommendations with mean target coverage of 67×.

Sequenced reads were aligned against the GRCh37/hg19 human reference genome using BWA-MEM^1^ v0.7.7, PCR duplicates were marked using Picard^2^ v1.136, and Genome Analysis Toolkit (GATK)^3,4^ v3.4-46 was applied for further BAM processing. Sample genotypes were called by GATK HaplotypeCaller algorithm, and all single gVCF-files were combined and jointly aggregated.

Samples were filtered based on high contamination (>5%), high proportion of chimeric alignment (>5%), low coverage (<20×), low call rate for SNVs (<99%), discordance with genome-wide array data (>5%) and mismatch between phenotypic and genotypic sex. Additional outlier samples (mean ±3 SD) were excluded based on the calculations of the number of variants, non-reference variants, heterozygous variants, heterozygous/homozygous variants ratio, transition/transversion ratio, insertion/deletion ratio for novel indels, and insertion/deletion ratio for indels observed in dbSNP. Extremely low coverage samples (below mean –3 SD) and extreme values (mean ±3 SD) of multiallelic SNVs and inbreeding coefficient F were removed additionally in WES quality control. After filtering and exclusion of duplicates between two datasets, the final WGS sample set contained 2420 and the final WES sample set 2356 unique samples.

The following filters were applied for genotype quality control: genotype quality <20, read depth >200 for WGS and <8 for WES, phred-scaled genotype likelihood of reference allele <20 for heterozygous and homozygous variant calls, and allele balance <0.2 or >0.8 for heterozygous calls. The GATK Variant Quality Score Recalibration (VQSR) was used to filter variants with a truth sensitivity of 99.8% for SNVs and of 99.9% for indels. Furthermore, variants with inbreeding coefficient less than –0.3, quality by depth <2 for SNVs and <3 for indels, call rate <90%, Hardy-Weinberg equilibrium P-value <1×10^-9^ were removed in addition to multi-allelic variants, and low-complexity regions^5^.

Sequence variants were annotated with Variant Effect Predictor^6^ version 87 (Gencode v19 on assembly GRCh37.p13) and ANNOVAR^7^.

**Identification of FH-associated variants**

We considered variants annotated as loss-of-function (*i.e*., premature stop codon, disruption of an essential splice site, or frameshift of the reading frame), deleterious missense variants in *LDLR,* gain-of-function missense variants in *PCKS9*, and deleterious variants in exon 26 (encompassing the binding site of the LDL receptor)^8^ in *APOB*. The following *in silico* prediction algorithms were considered for pathogenicity estimation of missense variants: SIFT^9^, PolyPhen-2 HumDiv^10^, CAROL^11^, CADD_Phred^12^, PhyloP^13^ and MutationTaster2^14^. The identified variants were further reviewed to estimate the carrier prevalence and prior association with hypercholesterolemia in the following databases: Genome Aggregation Database (gnomAD)^15^ v2.0, Sequencing Initiative Suomi (SISu)^16^, UCL low-density lipoprotein receptor variant database^17^, NCBI-ClinVar^18^, and public version of Human Gene Mutation Database^19^. A variant was considered as FH-associated if MAF <0.5%, identified in a carrier with LDL-C level of ≥4.0 mmol/L and 1) reported as pathogenic/likely pathogenic in NCBI-ClinVar^18^ and/or 2) determined pathogenic according to *in silico* prediction algorithms.

The determined untreated baseline LDL-C level threshold of ≥4.0 mmol/L considered in FH-associated variant identification corresponds to the lowest LDL-C value considered in the Dutch Lipid Clinic Network (DLCN) diagnostic criteria for familial hypercholesterolemia diagnosis^20^, suggested by the European Society of Cardiology and European Atherosclerosis Society for the management of dyslipidaemias^21^ (**Table S2**). While the LDL-C values measured with the conventional method and VAP were highly correlated in overlapping individuals (r=0.91; n=986) (**Figure S2**), the latter marginally underestimated the LDL-C parameter. An association between these parameters was modelled using linear regression:

$Metabolite_{C}\sim\mu+\beta*Metabolite_{VAP}+\varepsilon$,

where $Metabolite_{C}$ is the LDL-C values measured with the conventional method and $Metabolite_{VAP}$ is the LDL-C values measured with VAP.

The 4 mmol/L threshold established for the conventionally measured LDL-C value was scaled using the regression based coefficient to estimate the corresponding VAP-measured LDL-C value with $\hat{\beta}$ = 0.98 and $\hat{\mu}$ = 0.54. A conversion factor of 38.67 was used to convert the LDL-C mmol/L value to mg/dl. The corresponding VAP threshold was set at 136 mg/dl. In case LDL-C values were available from both platforms, the conventionally measured value was used. The effect of statin treatment in individuals, who had self-reported use at baseline or at appointment lipid measurement, was taken into account by dividing LDL-C value by 0.7, as implemented previously^22^ (termed "statin-adjusted"). The LDL-C level in four individuals carrying *APOB* variant p.Arg3527Gln or *LDLR* variant p.Arg115Cys, p.His250Arg or p.Gly396Ala did not exceed the determined LDL-C threshold. However, the former two have previously been associated with FH^23^ and are reported in NCBI-ClinVar^18^. The pathogenicity estimations based on *in silico* prediction algorithms of the latter two novel variants hinted at possible linkage with the disease and merited, therefore, further investigation (**Table S3, Table S4**).

**Phenotype-based inquiry of FH cases at EGCUT**

To ascertain whether any individuals had a record of clinically diagnosed FH at EGCUT, an additional search was conducted based on the phenotype data available within the biobank, collected at recruitment of the participants and linked EHRs, including national EHRs, hospitals’ electronic records and national health insurance fund database. No specific diagnosis code to differentiate FH from other forms of hypercholesterolemia existed in the International Classification of Diseases, Tenth Revision (ICD-10) prior to October 2016^58^. To delineate potential FH cases, hospital records and lipid measurements available at the Estonian Biobank were examined for the following: 1) a record of E78 with the term „familial hypercholesterolemia” documented in hospital clinical notes, and 2) LDL-C level of ≥4.0 mmol/L in conventional clinical, scaled VAP or scaled NMR measurements, statin-adjusted if on treatment. The regression-based coefficients to scale the NMR-based parameter were $\hat{\beta}$ = 1.57 and $\hat{\mu}$ = 0.46. Eight individuals, who did not have WGS/WES data available, met these criteria and were subjected to WES at EGCUT. The variant calling and quality control followed the same pipeline as described under WGS and WES sequencing and variant calling. Three out of eight carried FH-associated variants: two an *LDLR* variant p.Cys329Tyr and one an *APOB* variant p.Arg3527Gln.

**Management of probands**

Carriers of the FH-associated variants (probands) were contacted via regular mail and upon positive response scheduled for an initial appointment with a clinical genetics specialist (clinical geneticist and/or genetic counsellor) and clinical cardiologist at a cardiology clinic either at Tartu University Hospital or North Estonia Medical Centre (**Figure S1**). Contact information was retrieved from the Estonian Population Registry that is periodically linked with the Estonian Biobank data. Pre-appointment reports were formed based on phenotypic information retrieved from baseline data of the biobank collected at recruitment as well as from EHR for clinicians. The average time gap between recruitment to EGCUT and the initial appointment was 8.2 years.

At the initial appointment, probands were given the details of the project and signed an informed consent form. Subsequently, the family history of the participant of up to three generations (including the relative’s gender, age and main health issues and, in case of deceased individuals, the age and cause of death) was specified. Additionally, the medical history of the participant, regarding their health-related complaints, chronic and previous diseases, medications and lifestyle habits were collected. Prior statin prescriptions and duration of continuous use were asked in person, and prior prescriptions were additionally specified in the prescription database in EHR to ascertain adherence to statin treatment. Statin intensities were defined according to the 2013 American College of Cardiology/American Heart Association (ACC/AHA) Guideline on the Treatment of Blood Cholesterol to Reduce Atherosclerotic Cardiovascular Risk in Adults^24^. A standard clinical examination including ascertainment of features specific to FH (general status, state of the skin and visible mucosae, presence of oedema, tendon xanthomata, xanthelasmae and *arcus corneae*, auscultatory findings, heart rate, measurement of height, weight, waist and hip circumference, electrocardiogram and arterial blood pressure) followed. Finally, 50 ml of fasting blood from a peripheral vein was drawn for biochemical measurements and for a DNA-based confirmation of the genetic finding. The unused DNA and plasma were stored at the biobank.

Likelihood of FH was calculated using the "FH score", based on the guidelines in the Consensus Statement of the European Society^21^ according to the Dutch Lipid Clinic Network^20^ criteria. The score determines the likelihood of an FH diagnosis as unlikely (<3 points), possible (3-5), probable (6-8) or definite (>8) based on the individual’s cardiovascular disease (CVD) history, LDL-C level (statin-adjusted, if needed) at the initial appointment, physical symptoms (tendinous xanthoma, *arcus corneae*), family history of premature CVD in first degree relatives and presence of an FH-associated variant. The latter was considered in the score only if listed as pathogenic or likely pathogenic in NCBI-ClinVar (**Table S2**).

After the feedback, carriers with sub-clinical or clinical ASCVD were kept under further cardiovascular surveillance, and cases without disease manifestation were advised to monitor the lipid levels at their general practitioner's office. All probands were given recommendations for treatment and lifestyle modifications and referred to other specialists, if applicable. Next, the strategy of the screening for the condition, the essence of genetic testing, and privacy, confidentiality and implications of the results to the individual and his/her first- and second-degree relatives were explained. All probands were advised in person by the medical genetics specialist to inform family members of the medical management and implications of the study. They were provided with an information sheet of the study and biobank's contact information in written form for engagement of relatives to educate and encourage participation in cascade screening.

Relatives reported not to be residing in Estonia, or not able or willing to participate were excluded from further management (referred to as modified cascade). Thus, the number of expected cascades was adjusted during the project based on additional information received on relatives, relatedness between families of probands (adjusting for overlapping cascades), and results of genetic analyses of 1^st^ degree relatives for exclusion of non-carriers among 2^nd^ degree relatives. (**Table S5**).

**Management of cascade screening**

The investigation of the 1^st^ and 2^nd^ degree relatives invited to participate in cascade screening followed the same approach as for the probands. In case of low response rate within a family, the proband was called once by phone and specified the aim of cascade screening and the implications of participation. Only those individuals, who carried the variants identified in the family and without prevalent ASCVD, were subjected to instrumental investigations. All relatives were offered feedback on the specified genetic and clinical information and received recommendations for further clinical management at the feedback appointment, if needed (**Figure S1**).

REFERENCES

1. Li H, Durbin R. Fast and accurate short read alignment with Burrows-Wheeler transform. *Bioinformatics* 2009;**25**:1754–1760.

2. Picard. http://broadinstitute.github.io/picard/

3. Auwera GA Van Der, Carneiro MO, Hartl C, et al. From FastQ data to high confidence varant calls: the Genonme Analysis Toolkit best practices pipeline. *Curr Protoc Bioinforma* 2013:**43**:11.

4. McKenna A, Hanna M, Banks E, et al. The Genome Analysis Toolkit: A MapReduce framework for analyzing next-generation DNA sequencing data. *Genome Res* 2010;**20**:1297–1303.

5. Li H, Wren J. Toward better understanding of artifacts in variant calling from high-coverage samples. *Bioinformatics* 2014;**30**:2843–2851.

6. McLaren W, Gil L, Hunt SE, et al. The Ensembl Variant Effect Predictor. *Genome Biol* Genome Biology 2016;**17**:122.

7. Yang H, Wang K. Genomic variant annotation and prioritization with ANNOVAR and wANNOVAR. *Nat Protoc* 2015;**10**:1556–1566.

8. Borén J, Lee I, Zhu W, Arnold K, Taylor S, Innerarity TL. Identification of the low density lipoprotein receptor-binding site in apolipoprotein B100 and the modulation of its binding activity by the carboxyl terminus in familial defective Apo-B100. *J Clin Invest* 1998;**101**:1084–1093.

9. Ng PC, Henikoff S. SIFT: predicting amino acid changes that affect protein function. *Nucleic Acids Res* Oxford 2003;**31**:3812–3814.

10. Adzhubei IA, Schmidt S, Peshkin L, et al. A method and server for predicting damaging missense mutations. *Nat Methods* 2010;**7**:248–249.

11. Lopes MC, Joyce C, Ritchie GRS, John SL. Europe PMC Funders Group Europe PMC Funders Author Manuscripts A Combined Functional Annotation Score for Non-Synonymous Variants. *Hum Hered* 2012;**73**:47-51.

12. Kircher M, Witten DM, Jain P, O’Roak BJ, Cooper GM, Shendure J. A general framework for estimating the relative pathogenicity of human genetic variants. *Nat Genet* 2014;**46**:310–315.

13. Cooper GM, Stone EA, Asimenos G, Green ED, Batzoglou S, Sidow A. Distribution and intensity of constraint in mammalian genomic sequence. *Genome Res* 2005;**15**:901–913.

14. Schwarz JM, Cooper DN, Schuelke M, Seelow D. MutationTaster2: mutation prediction for the deep-sequencing age. *Nat Methods* 2014;**11**:361–362.

15. Lek M, Karczewski KJ, Minikel E V, et al. Analysis of protein-coding genetic variation in 60,706 humans. *Nature* 2016;**536**:285–91.

16. Sequencing Initiative Suomi project (SISu), Institute for Molecular Medicine Finland (FIMM), University of Helsinki, Finland. url: http://sisuproject.fi (1 January 2018)

17. Leigh S, Futema M, Whittall R, et al. The UCL low-density lipoprotein receptor gene variant database: pathogenicity update. *J Med Genet* 2017;**54**:217 LP-223.

18. Landrum MJ, Lee JM, Riley GR, Jang W, Rubinstein WS, Church DM, Maglott DR. ClinVar: public archive of relationships among sequence variation and human phenotype. *Nucleic Acids Res* 2014;**42**:D980–D985.

19. Stenson PD, Ball E V, Mort M, et al. Human Gene Mutation Database (HGMD): 2003 Update. *Hum Mutat* 2003;**21**:577–581.

20. Defesche JC, Lansberg PJ, Umans-Eckenhausen MA KJ. Advanced method for the identification of patients with inherited hypercholesterolemia. *Semin Vasc Med* 2004;**4**:59–65.

21. Catapano AL, Graham I, Backer G De, et al. 2016 ESC/EAS Guidelines for the Management of Dyslipidaemias. *Eur Heart J* 2016;**37**:2999–3058l.

22. Peloso GM, Auer PL, Bis JC, et al. Association of low-frequency and rare coding-sequence variants with blood lipids and coronary heart disease in 56,000 whites and blacks. *Am J Hum Genet* 2014;**94**:223–232.

23. Fouchier SW, Kastelein JJP, Defesche JC. Update of the molecular basis of familial hypercholesterolemia in The Netherlands. *Hum Mutat* 2005;**26**:550–556.

24. McClelland RL, Chung H, Detrano R, Post W, Kronmal RA. Distribution of Coronary Artery Calcium by Race, Gender, and Age. *Circulation* 2006;**113**:30 LP-37.
